# The T2-FLAIR mismatch sign in oncologic neuroradiology: History, current use, emerging data, and future directions

**DOI:** 10.1177/19714009231212375

**Published:** 2023-11-04

**Authors:** Samir A Dagher, Riley Hideo Lochner, Burak Berksu Ozkara, Donald F Schomer, Max Wintermark, Gregory N Fuller, F Eymen Ucisik

**Affiliations:** 1 Department of Neuroradiology, 4002 The University of Texas MD Anderson Cancer Center, Houston, TX, USA; 2 Section of Neuropathology, 4002 The University of Texas MD Anderson Cancer Center, Houston, TX, USA

**Keywords:** T2-FLAIR mismatch, radiogenomics, isocitrate dehydrogenase, astrocytoma, glioma, MRI

## Abstract

The T2-Fluid-Attenuated Inversion Recovery (T2-FLAIR) mismatch sign is a radiogenomic marker that is easily discernible on preoperative conventional MR imaging. Application of strict criteria (adult population, cerebral hemisphere location, and classic imaging morphology) permits the noninvasive preoperative diagnosis of isocitrate dehydrogenase (IDH)-mutant 1p/19q-non-codeleted diffuse astrocytoma with near-perfect specificity, albeit with variably low sensitivity. This leads to improved preoperative planning and patient counseling. More recent research has shown that the application of less strict criteria compromises the near-perfect specificity of the sign but remains adequate for ruling out IDH-wildtype (glioblastoma) phenotype, which bears a far grimmer prognosis compared to IDH-mutant diffuse astrocytic disease. In this review, we elaborate on the various definitions of the T2-FLAIR mismatch sign present in the literature, illustrate these with images obtained at a comprehensive cancer center, discuss the potential of the mismatch sign for application to certain pediatric-type brain tumors, namely dysembryoplastic neuroepithelial tumor and diffuse midline glioma, and elaborate upon the clinical, histologic, and molecular associations of the T2-FLAIR mismatch sign as recognized to date. Finally, the sign’s correlates in diffusion- and perfusion-weighted imaging are presented, and opportunities to further maximize the diagnostic and prognostic applications of the sign in the context of the 2021 revision of the WHO Classification of Central Nervous System Tumors are discussed.

## Introduction

The fully revised 5th edition (2021) of the WHO Classification of Central Nervous System Tumors (WHO CNS 2021) dramatically increases the number of tumor types that depend on molecular markers to establish a definitive diagnosis. This paradigm shift, which began with the WHO CNS 2000 and kept evolving over the subsequent revisions, stems from an inadequacy of histopathologic data alone to accurately predict clinical outcomes for patients with molecularly defined CNS tumors. Specifically, adult-type diffuse gliomas were immensely affected by this gradual paradigm shift and are now classified into three primary groups almost solely based on their molecular features: isocitrate dehydrogenase (IDH)-mutant, 1p/19q codeleted glioma (oligodendroglioma); IDH-mutant (IDHm), 1p/19q non-codeleted glioma (astrocytoma); and IDH-wildtype (IDHwt) glioma (glioblastoma).^1^ The classification update not only asserts the pivotal role of molecular data in the diagnosis and prognostication of CNS tumors but also highly incentivizes radiologists to further link imaging features with clinically critical genomic alterations, bolstering the importance of the emerging field of radiogenomics.

Significant advancements in computational methodologies, coupled with the growing importance of molecular data, have led to an exponential expansion of radiogenomics research. However, few radiogenomic applications have been translated into widespread clinical practice.^2^ Among those which were, a magnetic resonance imaging (MRI) sign termed “T2-Fluid-Attenuated Inversion Recovery (T2-FLAIR) mismatch” (T2FMM) stands out by virtue of its almost perfect specificity for the preoperative diagnosis of IDHm 1p/19q non-codeleted adult diffuse gliomas.^3^ This ability to preoperatively predict the IDHm non-codeleted phenotype allows improvement in patient counseling and pretreatment planning, as ongoing research suggests that a more aggressive surgical resection independently correlates with survival in this tumor type.^4–6^ Furthermore, it gives the surgeon more time for surgical planning, as the low-grade IDHm non-codeleted glioma is generally slow-growing in nature compared to its much more aggressive counterpart, the IDHwt glioma.^7^ However, the definition of this sign has been applied with variable levels of stringency over time, leading to some variability in the reported diagnostic accuracies and resulting in confusion among clinicians and researchers.^8–10^ Moreover, emerging data suggests potential alternative applications of the sign to other tumor types, further contributing to this confusion. In this article, we present a comprehensive overview of the various uses of the T2FMM sign, accompanied by sample cases, and suggest potential directions for future research.

## Astrocytoma in the 2021 update of the World Health Organization Classification of CNS Tumors

According to the WHO CNS 2021 classification, astrocytomas encompass diffusely infiltrating gliomas harboring two defining molecular features: (1) a mutation in isocitrate dehydrogenase 1 or 2 (IDH1/2; predominantly an IDH1 R132H substitution), diagnosed via immunohistochemistry or sequencing, and (2) absence of a simultaneous codeletion of the short arm of chromosome 1 and the long arm of chromosome 19 (1p/19q codeletion), preferentially detected by chromosomal microarray, array comparative genomic hybridization, genome-wide DNA methylation array, next-generation sequencing, or other reliable techniques over fluorescence in situ hybridization as the latter is prone to false positive results. Notably, loss of ATRX protein expression detected via immunohistochemistry can serve as a quick, widely available, and comparatively inexpensive surrogate for 1p/19q non-codeletion, as loss of ATRX expression is mutually exclusive with 1p/19q-whole arm codeletion. In this review, the term “codeleted” and “non-codeleted” will refer to 1p/19q codeletion status.

Astrocytomas range from CNS WHO grades 2 to 4. CNS WHO grade 2 and 3 astrocytomas lack microvascular proliferation, tumor necrosis, and CDKN2A/2B mutation (which can be detected via fluorescent in situ hybridization, next-generation sequencing, and a number of other techniques) compared to CNS WHO grade 4 astrocytomas, which require the presence of at least one of these three histo-molecular attributes.^11,12^ Tumor grade has been correlated with patient survival. While the average survival is more than 10 years for CNS WHO grade 2 astrocytomas, it remains around 3 years for CNS WHO grade 3 tumors.^13^ Without accounting for these grades, the overall survival falls between that of oligodendroglioma, which has a better survival, and that of glioblastoma, which has a much poorer survival.

## History, definition, sensitivity, and specificity of the T2-FLAIR mismatch sign

The concept of “T2-FLAIR suppression” was first described in 2011 by Tay and colleagues, who reported the phenomenon in a series of eight protoplasmic astrocytomas, a historical entity that was subsequently removed from the WHO CNS 2016 revised 4th edition, with its members included within the IDHm astrocytoma rubric.^14^

The T2FMM, as originally correlated by Patel and colleagues in 2017 with the IDHm non-codeleted phenotype, was defined as the “presence of complete/near-complete hyperintense signal on T2-weighted images (T2WI), and relatively hypointense signal on FLAIR except for a hyperintense peripheral rim.” In their retrospective cohort, they identified the T2FMM with a specificity of 100% for the diagnosis of IDHm non-codeleted diffuse glioma in both the training and the validation sets, comprising 125 and 82 lower-grade gliomas (LGGs), respectively. However, the sensitivity of the sign remained low at 22.1% and 45.5%, respectively.^3^

Later, as some studies applied the sign with less stringency, these strict criteria were sometimes referred to as the “classic” criteria of T2FMM in an effort to prevent confusion.^15^ In this review, we will also use this descriptor while referring to the strict (“classic”) T2FMM criteria.

The “classic” T2FMM was later validated by several retrospective cohorts that consistently yielded a near-perfect specificity, but with a variably low sensitivity. A multicenter retrospective cohort of 154 nonenhancing LGGs by Broen and colleagues found that strict application of the criteria yielded a specificity of 100% and sensitivity of 51%.^16^ Batchala and colleagues also found 100% specificity and positive predictive value of the T2FMM for the detection of 1p/19q non-codeleted status in a cohort of 106 IDHm LGGs. These findings rendered this sign superior to several other metrics tested in the same study, such as texture, T2* susceptibility, enhancement, cyst presence, necrosis, and tumor location.^17^

A systematic review and diagnostic meta-analysis by Han and colleagues quantitatively assessed the diagnostic performance of T2FMM across 11 studies (13 cohorts) aggregating 1933 patients. It showed a pooled sensitivity and specificity of 42% (95% confidence interval [CI]: 34–50) and 99% (95%CI: 96–100), respectively, of the sign to diagnose IDHm non-codeleted LGGs, as well as a moderate-to-almost-perfect inter-rater agreement, with Cohen’s kappa = 0.56 to 0.89.^9^ Another meta-analysis of 14 cohorts aggregating 1736 patients yielded a pooled sensitivity and specificity of 40% (95%CI: 31–50) and 97% (95%CI: 93–99), respectively.^18^ Additionally, in their meta-analysis of 8 studies (10 cohorts) including 1342 patients, Do and colleagues computed a pooled sensitivity of 40% (95%CI: 28–53) and a pooled specificity of 100% (95% CI: 95–100).^8^ Notably, several systematic reviews pointed at the high heterogeneity present amongst the demographics and methodologies of the included studies, such as the variable definition of the T2FMM and MRI acquisition parameters.^8–10^

## The T2-FLAIR mismatch sign as a tool for diagnosing IDH-mutant non-codeleted gliomas

**Figure 1.**
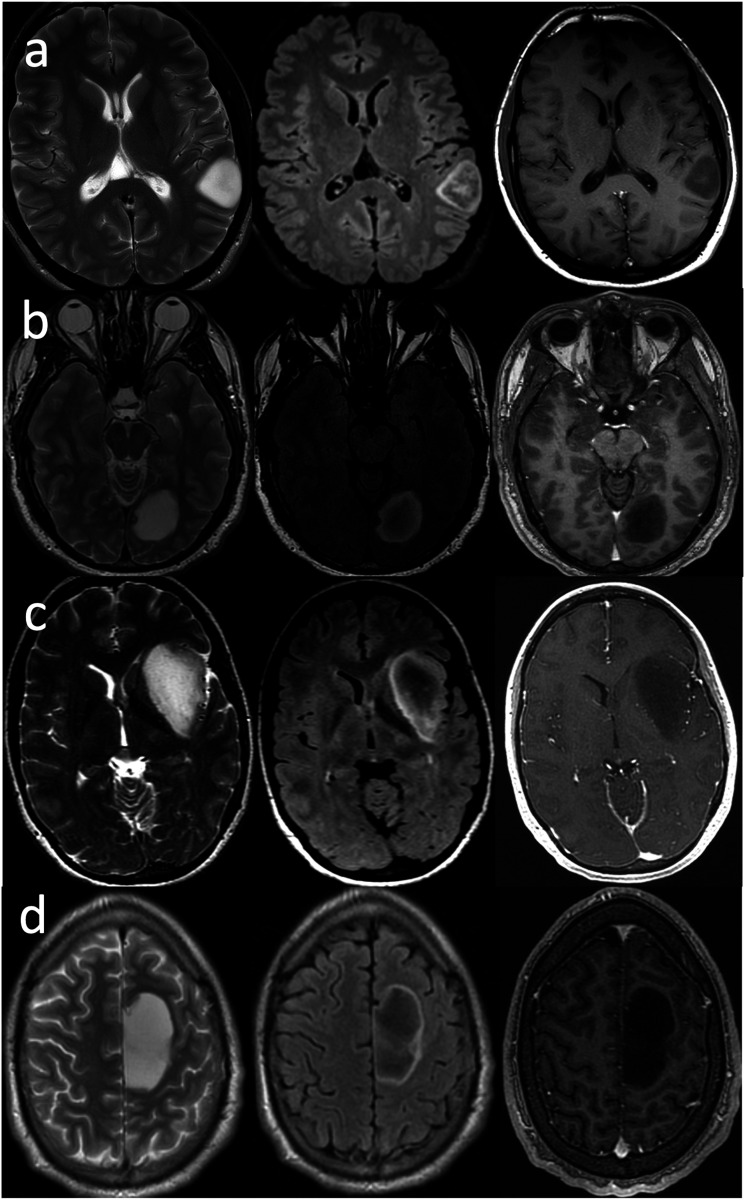
IDH-mutant, 1p/19q non-codeleted diffuse gliomas in two 37-year-old males (a), (b), a 31-year-old female (c), and a 31-year-old male (d). All demonstrate the strict criteria of T2FMM: complete or near-complete homogeneous hyperintensity on T2WI (left column), relatively hypointense signal on FLAIR, except for a thin hyperintense peripheral rim (middle column), and little-to-no enhancement on postcontrast T1WI (right column). Of note, tumor “a” exhibits an intrinsic central hyperintensity on the pre-contrast T1WI (not shown) and does not enhance.

Diagnosing IDHm non-codeleted gliomas was the original purpose of the T2-FLAIR mismatch sign when it was first introduced in 2017. In their 2020 review article, Jain and colleagues, who were among the researchers originally defining the sign, underscored that the variability in the “real-world” implementation of T2FMM might have interfered with its widespread clinical adoption.^7^ The confusion arose due to the discrepancy between the initial studies, which achieved nearly 100% specificity for IDHm non-codeleted status using stringent criteria, and subsequent research which employed a more “lenient” approach, resulting in diminished specificity. To mitigate this confusion, they provided the following inclusion criteria, to be strictly applied in a stepwise fashion:

1. Complete or near-complete homogeneous hyperintensity on T2WI. This was defined as a strikingly diffuse high signal on T2WI, with only a few subtle areas of hypointensity. However, relative hypointensity due to entrapped gray matter is acceptable and should resolve upon radiologic examination in a different plane.
2. Relatively hypointense signal on FLAIR, except for a thin hyperintense peripheral rim. This was defined as a hypointense signal of the entire lesion relative to T2WI, except for a thin peripheral hyperintense rim, which may not necessarily be complete. The extent of suppression may be inhomogeneous (Figure 1).

**Figure 2.**
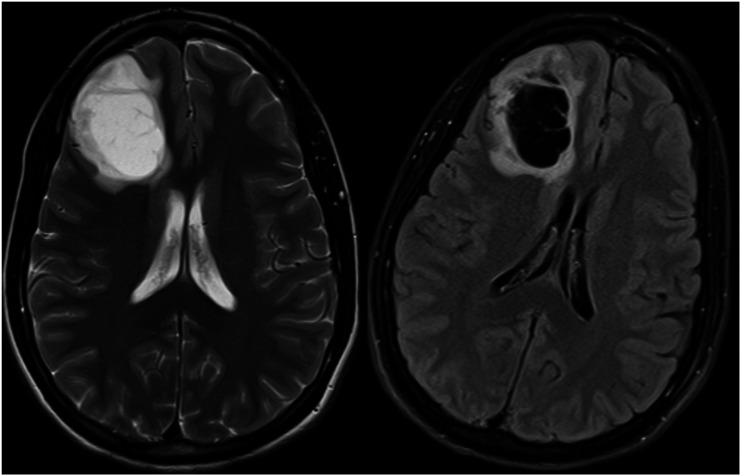
IDH-mutant, 1p/19q non-codeleted glioma in a 25-year-old male. The tumor harbors a large cystic component that follows the cerebrospinal fluid signal on the T2WI (left) and FLAIR (right), which does not qualify as T2FMM.

The authors also emphasized that FLAIR suppression within fluid-containing intra-tumoral macrocysts and necrotic areas does not qualify as T2FMM (Figure 2), and that T2FMM should only be described in lesions that demonstrate little-to-no contrast enhancement. Even a small enhancing nodule would disqualify the lesion from fulfilling the strict criteria. In the situation of a non-contrast enhanced study, the authors suggested using the T2-weighted and FLAIR sequences to predict the presence of an enhancing component, as enhancing tumor components are usually hypointense on T2WI and hyperintense on FLAIR.^7^ Some studies have discussed that T2FMM is prone to inherent inter-rater and even intra-reader variability, even among experienced readers, since one of the core criteria, “strikingly diffuse high signal on T2WI with merely few subtle areas of hypointensity” is by nature somewhat subjective and may depend on what the reader considers as “subtle.”^3,19^

Some studies adopted less stringent versions of these criteria, yet still achieved perfect or near-perfect specificity for IDHm non-codeleted gliomas, possibly due to relatively small sample sizes and heterogeneous sample populations. In their retrospective cohort of 59 LGGs, Lasocki and colleagues found 100% specificity and 37% sensitivity of the sign for IDHm non-codeleted gliomas, even though they also included lesions displaying mismatch in their subsections, as long as those subsections comprised more than 50% of the lesion.^20^ Foltyn and colleagues studied T2FMM in a large retrospective cohort of 408 patients with diffuse glioma. While they adhered to the strict T2WI and FLAIR criteria, contrast-enhancing lesions were not excluded. In addition, unlike previous studies, their cohort included not only low-grade but also high-grade gliomas. Despite the slightly more “relaxed” application of the sign, and inclusion of higher-grade tumors, they obtained 100% specificity and 22% sensitivity for IDHm non-codeleted gliomas. Of note, the enhancing tumor volume in mismatch-positive cases was very small (interquartile range below 0.5 cm^3^). These findings suggest that the presence of a small enhancing tumor burden may not necessarily compromise the sign’s near-perfect specificity.^21^ In their institutional training and validation cohorts of 585 LGGs with similar numbers of IDHm codeleted and non-codeleted tumors, Li and colleagues published in 2022 the diagnostic performance of the “hyperFLAIRrim” sign. It was defined as a hyperintense FLAIR rim enclosing a relatively hypointense core, irrespective of T2WI homogeneity. Unlike with “classic” T2FMM, the absence of enhancement or cystic components was not sought. While both the “classic” T2FMM sign and the “hyperFLAIRrim” sign were able to detect IDHm non-codeleted status with perfect specificity, the former had a sensitivity of 53.9%, which increased to 71.3% with the use of the latter. However, it is important to note that the findings of this study are validated only for nonenhancing or mildly enhancing tumors, as tumors with ring-enhancement or evident enhancement and necrosis were excluded.^19^

Using 105 LGGs from the TCGA/TCIA multicenter cohort, Mohammed and colleagues developed a fully automated algorithm to quantify the extent of T2FMM through geographically weighted regression. This method not only replicated the near-perfect specificity for IDHm non-codeleted status, but also greatly increased the sensitivity of T2FMM to 98%, while offering protection from intra- and inter-reader variability. This quantification method remains to be tested and validated in the real-world environment.^22^

Despite being extensively studied in the preoperative setting, the T2FMM sign remains poorly studied in the post-treatment setting. Throckmorton and colleagues observed that after treatment (radiotherapy with or without chemotherapy), all 10 of their originally mismatch-positive residual tumors showed progressive “fading” of mismatch due to increasing FLAIR signal, suggesting that changes in T2FMM status may reflect treatment response. Additionally, T2FMM was observed in five of eight originally mismatch-positive tumors treated with gross total resection which subsequently recurred.^15^

## The “partial” T2-FLAIR mismatch sign as a tool for assessing IDH-wildtype status

**Figure 3.**
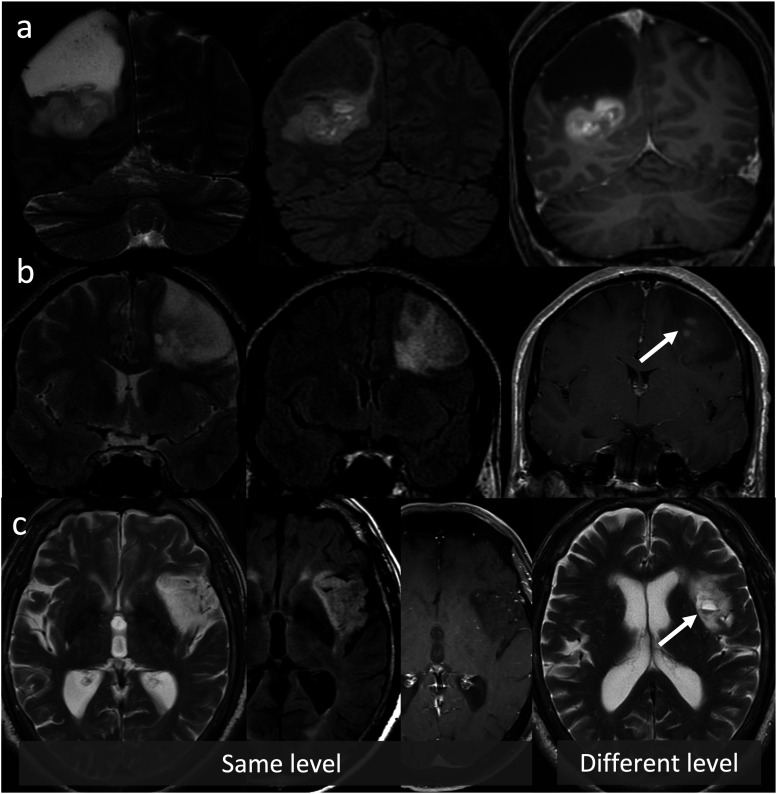
Lesions that show T2FMM in their subregions. (a) IDH-mutant, 1p/19q non-codeleted glioma in a 43-year-old female. While the superior subregion of the mass demonstrates T2FMM, the inferior subregion has heterogenous signal on T2WI (left column), lacks suppression on FLAIR (middle column), and diffusely enhances (right column). (b) IDH-mutant, 1p/19q codeleted glioma with partial T2FMM in a 46-year-old male. While the superficial and peripheral subregions demonstrate T2FMM, the deeper and more central portion demonstrates heterogenous signal on T2WI, lacks suppression on FLAIR, and shows patchy enhancement (arrow). (c) IDH-mutant, 1p/19q codeleted glioma with partial T2FMM in an 81-year-old male. While the more inferior subregion demonstrates T2FMM, the more superior subregion shows small cysts, one of which has dependent-layering susceptibility compatible with blood products (arrow).

**Figure 4.**
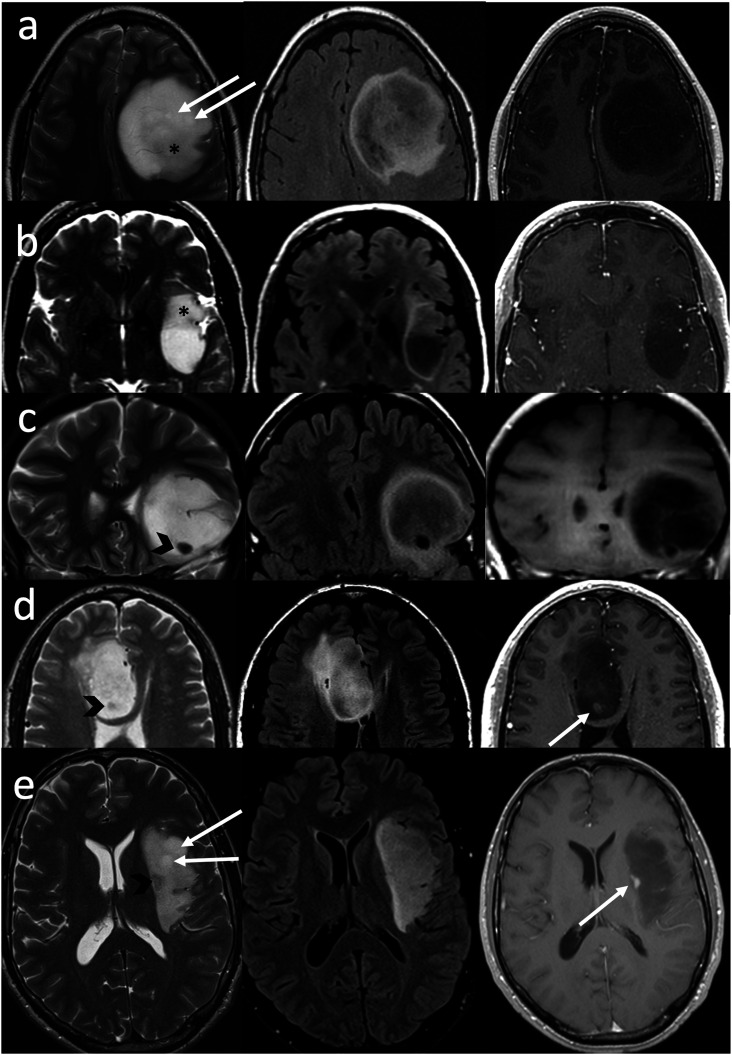
IDH-mutant, 1p/19q non-codeleted gliomas in a 26-year-old female (a), a 43-year-old female (b), a 36-year-old male (c), a 52-year-old male (d), and a 42-year-old male (e). The lesions do not show the “classic” T2FMM sign, as they meet the FLAIR criteria but not the T2WI criteria. On T2WI (left column), they lack “complete homogeneous hyperintensity” due to small macrocystic components (arrows, left column) or relatively hypointense regions/foci (asterisks and arrowheads, left column). All lesions meet the FLAIR criteria, as they demonstrate relatively hypointense signal except for a thin peripheral rim (middle column). As mentioned, homogenous signal is not a requirement for the FLAIR sequence like it is for the T2WI. This pattern (heterogeneous T2, hyperintense FLAIR rim) has been described as the “geographic T2FMM sign” by Throckmorton and colleagues.^15^ Note that some lesions (d)–(e) show small enhancing nodules (arrows) also disqualifying them from meeting the strict criteria.

When the radiologist's objective is primarily to assess the IDH mutation status rather than the 1p/19q codeletion status, recent studies indicate a more inclusive, that is, more lenient application of the sign could be a viable approach.^7^ This approach includes lesions that demonstrate T2FMM only in their subregions (Figure 3), or lesions that fulfill the FLAIR but not the T2WI criteria (Figure 4). This phenomenon has been referred to as “modified,” “partial,” or “relaxed” T2FMM in the literature.^23,24^

In their multicenter retrospective cohort published in 2019, Juratli and colleagues were the first to report a less-than-perfect specificity of 76%, with not only 60 of 82 IDHm non-codeleted astrocytomas, but also 12 of 42 IDHm codeleted oligodendrogliomas, exhibiting T2FMM. Significantly, however, no IDHwt gliomas exhibited the sign.^25^ A more “relaxed” version of the sign was adopted in this study, with the inclusion of some lesions with relatively heterogenous hyperintensity on T2WI, incomplete suppression of FLAIR signal, or areas of enhancement. This compromised the specificity of the radiogenomic marker for 1p/19q codeletion status, but not to IDH mutation status.^7^

Throckmorton and colleagues evaluated in 2020 the performance of a more “relaxed” application of T2FMM in a retrospective cohort of 64 histologically proven low-grade astrocytomas as per the WHO CNS 2016 criteria. Of these, 53 had known IDH status (47 IDHm and 6 IDHwt). Even though some relevant molecular features were not available, such as the 1p/19q codeletion or ATRX expression status, no histologic oligodendrogliomas were included. The patients were classified into three groups: those that demonstrated “classic” T2FMM, those that fulfilled the FLAIR criterion but demonstrated heterogenous rather than homogeneous hyperintensity on T2WI, and those that showed no mismatch at all. The second category was referred to as the “geographic mismatch” group (Figure 4). With the inclusion of cases with “geographic mismatch,” the sensitivity for IDHm status increased from 34% to 74%, while the specificity remained at 100% (numbers calculated by the authors of the present review article using the data provided in the referenced study). Of note, half of the cases with “geographic” T2FMM had enhancing components.^15^ One may argue that the perfect specificity with these more “relaxed” criteria may not have been preserved if oligodendrogliomas were included in the study. However, the findings do emphasize that the stringency of the applied criteria depends on the clinical question posed, and that using the more “relaxed” criteria may be better suited for IDH mutation status determination.

In 2021, another retrospective cohort of 199 high-grade gliomas found that “fluid attenuation in the nonenhancing component” was a significant independent predictor of the IDHm status. “Fluid attenuation in the nonenhancing component” was defined as any tumor volume with hyperintense T2 signal accompanied by a corresponding hypointense FLAIR, excluding any ring- or rim-enhancing region (e.g., necrotic core) or vasogenic edema. Therefore, in addition to lesions with “partial” T2FMM, those with cystic nonenhancing component(s) were also considered for “fluid attenuation.” The latter phenomenon was present in 11 out of 16 IDHm, and in 3 out of 183 IDHwt high-grade gliomas (*p*-value <.001).^24^ Although IDHwt status was not completely excluded, this finding strongly suggested an IDHm status.

Most recently, a large multi-institutional retrospective cohort study of 2165 WHO grade 4 gliomas identified the “partial” T2FMM in 32 of 121 IDHm, and in 8 of 2044 IDHwt gliomas, yielding a sensitivity of 26.4%, specificity of 99.6%, positive predictive value of 80.0%, and negative predictive of 95.8% for the detection of IDHm status. Thus, for tumors exhibiting the “partial” T2FMM sign, the likelihood of IDHwt status was significantly decreased, yet not completely excluded. When the analysis was repeated for “fluid attenuation in the nonenhancing component” (i.e., when both lesions with T2FMM and those with cystic components were considered), the sensitivity for IDHm status increased (40.5%) at the expense of the positive predictive value (49.5%).^23^

## Suggested uses in pediatric central nervous system tumors

**Figure 5.**
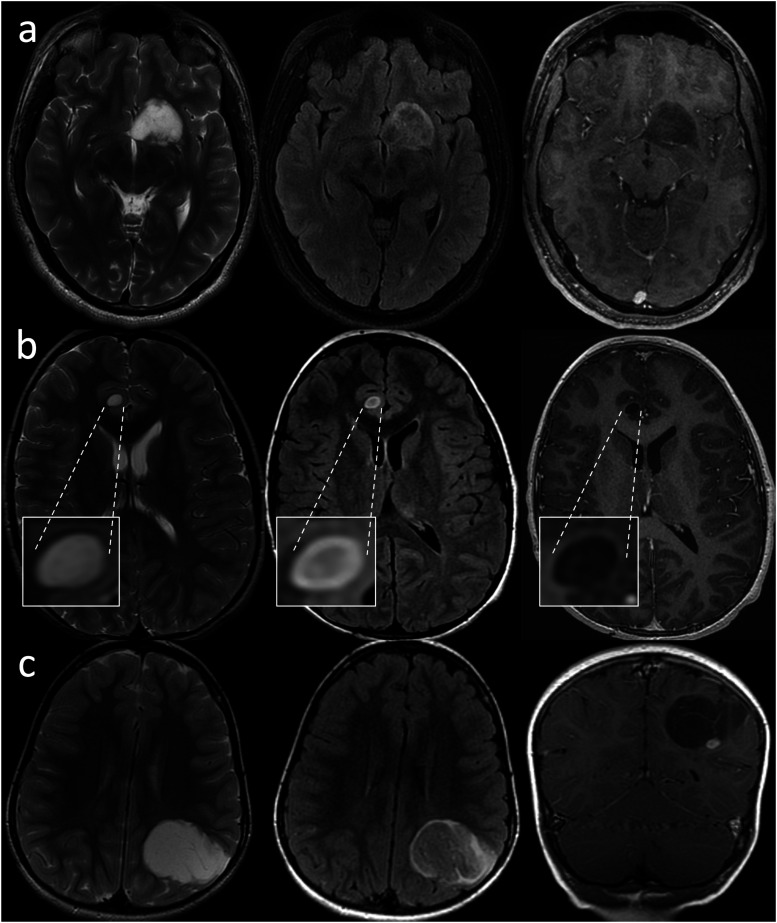
Dysembryoplastic neuroepithelial tumor (DNET) in a 19-year-old male (a), a 6-year-old male (b) and a 5-year-old female (c), diagnosed either via biopsy or resection. (a), (b): The tumors fulfill the strict criteria of the T2FMM sign: complete or near-complete homogeneous hyperintensity on T2WI (left column), relatively hypointense signal on FLAIR except for a thin hyperintense peripheral rim (middle column), and little-to-no enhancement on the postcontrast T1WI (right column). (c): The tumor only partially fulfills the criteria. While the T2WI and FLAIR criteria are met, this lesion has an enhancing nodule as seen on the coronal postcontrast T1WI.

Phenomena similar to T2FMM have also been reported in several dysembryoplastic neuroepithelial tumor (DNET) cases (Figure 5). DNETs are pediatric-type glioneuronal tumors characterized by medically refractory epilepsy, and typically present as cortical lesions with little-to-no contrast enhancement on MR imaging.^26^ In a 2007 case-control series consisting of DNETs and control cases with other CNS tumors considered in the differential diagnosis of DNETs (oligodendroglioma, low-grade astrocytomas, and gangliogliomas), Parmar and colleagues observed a thin high-FLAIR rim on MRI surrounding 9 of 11 DNETs, as opposed to 2 of 21 control cases. They named this imaging feature the “FLAIR ring sign.” Upon histopathologic correlation, they attributed this observation to the presence of “loose peripheral glioneuronal elements” surrounding a central cystic component attenuated by inversion recovery.^27^ In another study from 2020, Onishi and colleagues also observed a similar phenomenon among 8 of 11 DNETs in their institutional cohort. They discussed that most studies of T2FMM only included LGGs, possibly introducing selection bias and contributing to the perfect specificity of the T2FMM.^28^

**Figure 6.**
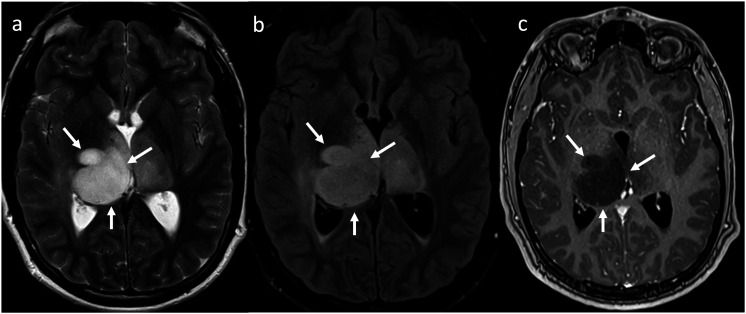
H3K27-altered diffuse midline glioma with partial T2FMM in a 19-year-old male. The portion of the mass that involves the right thalamus and basal ganglia (arrows) demonstrates mismatch, evidenced by homogenously bright signal on the T2WI (a), relatively hypointense signal on FLAIR except for a thin hyperintense peripheral rim (b), and lack of enhancement on postcontrast T1WI (c). The remainder of the lesion lacks mismatch, as it has lower signal on T2WI and lacks suppression on FLAIR sequence.

Although originally exclusively defined for adult-type diffuse gliomas, emerging data suggest the potential use of T2FMM in the pediatric-type diffuse glioma population (Figure 6). A recent retrospective cohort study suggested this sign is significantly more common in H3K27-altered compared to H3K27-wildtype diffuse midline gliomas.^29^ Another retrospective cohort consisting of 21 patients with diffuse intrinsic pontine glioma (now under H3K27-altered diffuse midline gliomas in the WHO CNS 2021 revision)^1^ identified some degree of T2FMM in five patients. These patients had significantly better overall survival than their T2FMM-negative counterparts who received the same treatment, suggesting that the presence of “partial” T2FMM may be an indicator of better response to focused fractionated radiotherapy in this patient group.^30^

Most recently, in their much larger institutional cohort of 349 histologically proven pediatric LGGs, Wagner and colleagues identified the T2FMM sign in 25 lesions (7.2%), confirming that the T2FMM is seen in a considerable number of pediatric gliomas. In particular, the sign was present in 9 of 24 DNETs, 5 of 22 diffuse astrocytomas, 1 of 5 glioneuronal tumors, 1 of 5 angiocentric gliomas, 8 of 66 low-grade astrocytomas, and 1 of 151 pilocytic astrocytomas.^31^

Though overlap is present, lesion location can aid in the differential diagnosis of pediatric and young adult brain lesions that demonstrate T2FMM. IDHm non-codeleted gliomas can be seen anywhere in the CNS but are most commonly supratentorial and usually centered within or near the frontal lobes. DNETs are cortical-based tumors that arise in any part of the cerebral cortex, but they show a predilection for the mesial temporal lobes and the frontal lobes. Diffuse midline gliomas occur in the brainstem or thalami, though they can arise in the spinal cord in adolescents or adults.

## False positive cases

To the best of our knowledge, only two “false positive” cases of “classic” T2-FLAIR mismatch have been reported within the specific patient population for which the T2FMM was originally defined (adult patients with imaging and clinical findings suggestive of an infiltrating glioma). The cases were an IDHm codeleted glioma in a 44-year-old male,^32^ and an IDH1/2 wildtype glioma in a 57-year-old male.^33^ The remaining “false-positive” case reports were either a product of a “relaxed” application of the sign or were outside the adult glioma group.

Even though it is difficult to pinpoint the cause of “false positivity” in most adult glioma studies because the individual cases’ imaging findings are not available for review, meta-analyses suggest that these “false positives” may be a product of heterogeneous application of the T2FMM criteria ranging from strict to “relaxed.” This was suggested by a systematic review and meta-analysis by Park and colleagues which showed a tradeoff between the sensitivity and specificity of the included studies.^10^ Significant heterogeneity was also observed in subsequent meta-analyses.^8,9,18^ In addition, some “false positive” cases may be secondary to MRI acquisition techniques or interpretation errors. For example, a 2019 cohort described T2FMM not only in IDHm non-codeleted gliomas but also in IDHm codeleted and IDHwt gliomas, leading to a significantly lower specificity of 72.5% compared to those previously described. Due to this discrepancy, the authors revisited the respective “false positive” cases in their series and subsequently acknowledged the possible confounding variable of background signal attenuation on the FLAIR sequences in some cases, and incorrect assignment of the sign to a cystic component in another case. The authors postulate that such inaccuracies may reflect the real-life vulnerability of the sign.^34^

To the best of our knowledge, the remaining “false positive” cases are described in pediatric and young adult patients, thus comprising a younger population than the one studied by Patel and colleagues in 2017.^3^ In addition, this group includes pediatric-type gliomas, such as pilocytic and pilomyxoid astrocytoma, diffuse midline gliomas H3K27-altered, MYB/MYBL1-altered diffuse astrocytoma, low-grade astrocytoma, diffuse astrocytoma, angiocentric glioma, DNET, other glioneuronal tumors, as well as gray matter heterotopia. Nevertheless, awareness of this is important, as some pediatric tumors exhibiting mismatch may first manifest in young adulthood and may have overlapping lesion location, MRI characteristics, and clinical presentation in common with those features seen in IDHm non-codeleted glioma. Due to this overlap, caution should be exercised when applying T2FMM in older children and in younger adults.^27,28,31,32,35^

## Clinical, histopathologic, and molecular associations

Only a few studies limited by small sample size have addressed the clinical, histopathologic, and molecular correlates of the T2FMM to date. Histo-molecular characterization of tumors has also been limited by intraoperative sampling errors compounded by the heterogeneous nature of gliomas.

Broen and colleagues found no statistically significant differences in sex, age at diagnosis, tumor location, and WHO CNS 2016 grade between T2FMM-positive and negative cases.^16^ They also had similar presenting symptoms and extents of surgical resection.^36^ Survival analysis performed in multiple studies found no statistically significant differences in progression-free survival and overall survival in patients with IDHm non-codeleted mismatched versus non-mismatched tumors.^3,25,29,37–39^ Similarly, the “partial” T2FMM sign did not confer a statistically significant survival advantage in high-grade gliomas of the ReSPOND cohort, although a nonsignificant trend towards slightly longer survival was observed.^23^

Patel and colleagues performed a histopathologic review of 30 IDHm non-codeleted LGGs from the TCGA/TCIA cohort, 15 of which displayed T2FMM on MRI, and found a trend for the presence of microcysts in T2FMM-positive cases. The trend did not reach statistical significance, possibly due to the small sample size and heterogeneous quality and consistency of the tissue samples, as discussed by the authors.^3^ Later, in a retrospective cohort of 64 LGGs, Deguchi and colleagues found a statistically significant association between microcysts on histopathology and the T2FMM sign. In one of the patients with an IDHm non-codeleted glioma and “partial” T2FMM, targeted multicentric sampling was performed and revealed abundant microcysts in the T2-FLAIR mismatched regions, but only scarce microcysts in the matched regions.^38^ Similarly, Fujita and colleagues reported in their institutional series of 17 IDHm non-codeleted tumors that the core of mismatched astrocytomas had multiple microcysts of variable size, compared to abundant neuroglial fibrils and cellularity in their periphery, as well as in the core of mismatch-negative gliomas.^40^ In addition to redemonstrating the association between T2FMM and microcystic change, Yamashita and colleagues showed in their recent retrospective series of 36 IDHm non-codeleted gliomas that mismatch-positive tumors were associated with significantly larger intercellular spaces than their mismatch-negative counterparts, even in the absence of overt microcyst formation. These findings suggest that enlarged intercellular spaces could partly be responsible for the T2FMM and may also explain why microcysts are not observed in all mismatched cases.^39^

Several retrospective cohorts have attempted to uncover possible molecular associations with T2FMM. To the best of our knowledge, upregulation of the mTOR pathway is the only association identified to date. When they first described the mismatch sign in 2017, Patel and colleagues conducted an exploratory analysis of the 125 LGGs from the TCGA/TCIA database for possible molecular associations with T2FMM. Even though their gene expression profile and proteomic analyses revealed that mTOR pathway upregulation was significantly more common in mismatch-positive LGGs than in mismatch-negative LGGs, the significant difference did not persist when the analysis was restricted to IDHm non-codeleted gliomas.^3^ However, in a later study, Yamashita and colleagues observed the aforesaid difference with statistical significance in a smaller series of 18 IDHm non-codeleted LGGs.^39^ As recent research suggests that mTOR pathway upregulation may contribute to tumor aggressiveness, and thus may serve as a potential treatment target,^41^ this potential association may be worth exploring. Attempts to uncover other molecular associations have not yet revealed statistically significant results. These include glioma-related gene mutation analysis interrogating the *PDGFRA, EGFR, PTEN, CDK4, MDM2, NFKB1A, TP53,* and *CDKN2A* genes; DNA copy number analysis; DNA methylation analysis; and MGMT promoter methylation status analysis.^3,24,36,39^

A recent large pediatric cohort provided descriptive data regarding the molecular profiles of pediatric LGGs with T2FMM. While the T2FMM was absent in tumors with more common molecular alterations such as KIAA1549-BRAF–fused and BRAF p. V600E–mutated LGGs, it was more commonly noted in tumors with rare molecular markers such as MYBL1, MYB, FGFR1-TKDD, FGFR1-TACC1, FGFR4, GOPC-ROS1, IDH1, MET N375S, MYB-QKI, and PDGFB-LRP1.^31^

## The T2-FLAIR mismatch sign and diffusion- and perfusion-weighted imaging

Several retrospective studies have explored the association of T2FMM with diffusion-weighted and perfusion-weighted imaging techniques.

It was suggested that the mismatched cores of IDHm non-codeleted gliomas are associated with significantly higher mean ADC values compared to both their FLAIR hyperintense rim, as well as to other mismatch-negative IDHm gliomas.^14,21,40,42^ The authors attributed the higher ADC values to more readily occurring facilitated free water diffusion within the microcyst-rich core of the lesions. In their institutional training (*n* = 134) and external validation (*n* = 93) cohorts of histopathologically low-grade IDHm codeleted, IDHm non-codeleted, and IDHwt gliomas, Aliotta and colleagues identified an optimal threshold of 0.35 for the fractional tumor volume with ADC >1.5  ×  10^−3^ mm^2^/s for the detection of IDHm non-codeleted gliomas. This threshold value yielded a sensitivity/specificity of 0.57/0.93 and 0.57/0.91 in the training and validation data sets, respectively. The sensitivity/specificity of the “classic” T2FMM in these datasets were 0.17/1.0 and 0.15/1.0, respectively. An “either/or” approach for the two imaging features yielded a sensitivity/specificity of 0.65/0.93 and 0.59/0.91, respectively. Thus, combining the features greatly increased the sensitivity compared to solely using T2FMM, at the cost of slightly compromising the perfect specificity. Collectively, 4 of the 45 patients with IDHwt glioma in the study were misidentified as IDHm non-codeleted glioma. Notably, 3 of these 4 patients were younger patients in their 30 s and had a more favorable clinical course compared to the remaining patients with IDHwt gliomas, although the survival benefit in this small sample did not reach statistical significance.^37^ The clinical use of this approach may call for caution, and future studies may focus on further refining the application.

In a retrospective cohort study, Foltyn and colleagues correlated the presence of T2FMM with dynamic susceptibility contrast imaging in 75 IDHm gliomas. They found that IDHm gliomas with mismatch had significantly lower relative cerebral blood volume (rCBV) values than those without mismatch (median rCBV of 1.81 vs 1.22). The trend persisted when the analysis was limited to the non-codeleted subgroup; however, the difference became smaller (median rCBV of 1.52 vs 1.22) and lost its statistical significance. Further studies are needed to determine the clinical utility of this trend.^21^

## Suggested directions for future research

T2FMM permits the non-invasive and confident rule-in of the IDHm non-codeleted phenotype owing to its near-perfect specificity and ease of clinical application. The preoperative information provided by this sign, using widely available conventional MRI sequences, improves surgical planning and patient counseling.^3,7^

**Figure 7.**
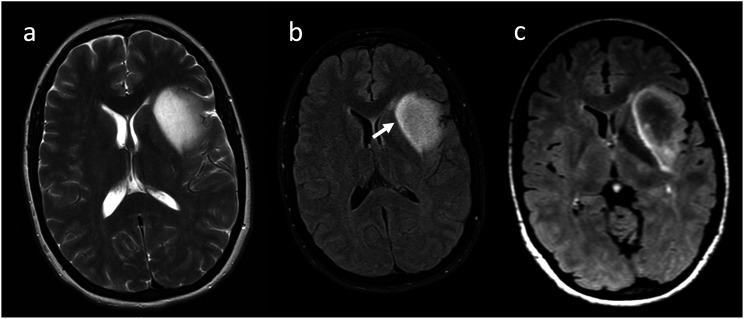
(a),(b) IDH-mutant, 1p/19q non-codeleted glioma with T2FMM in a 30-year-old female. T2WI (a) shows a left insular mass with homogenous T2 signal, and the FLAIR sequence (b) shows subtle hypointense signal relative to a thin peripheral hyperintense rim. The hyperintense rim is only appreciated on the medial aspect of the lesion (arrow). (c) 3 weeks later a repeat MRI was performed using a different scanner. The relative hypointensity is much more evident on this study, and the thin hyperintense rim is circumferential. The acquisition information for the images “b” versus “c” were as follows: magnetic field strength of 1.5 versus 3 T, Echo Time of 124 versus 91 ms, Inversion Time of 2500 versus 2098 ms. Image “b” was obtained via 2D acquisition with fat saturation, and image “c” via 3D acquisition without fat saturation.

Several factors lead to some heterogeneity in our existing knowledge. While variable interpretation of the sign’s definition in different studies introduced some confusion, it also led to the discovery of potential alternative uses, such as the effective exclusion of IDHwt gliomas when the “relaxed” application is employed. Recent changes in the WHO CNS classification have contributed to the heterogeneity of retrospectively collected data. This retrospective collection might have also rendered the studies vulnerable to heterogeneities resulting from MRI acquisition techniques (particularly field strength and inversion times), which have been shown to affect the degree of T2WI signal homogeneity, and even occasionally mask its characteristic FLAIR appearance (Figure 7).^16,20,34,43^ Future prospective studies with large sample sizes, standardized MRI acquisition parameters, standardized T2FMM nomenclature, and updated diagnosis and grading according to the WHO CNS 2021 classification could help minimize these heterogeneities and increase accuracy. To standardize mismatch labeling across studies, automated algorithms quantifying the extent of mismatch with high accuracy could be used.^22^ Upcoming studies could include patients with suspected rather than histopathologically confirmed gliomas to achieve a more reliable diagnostic accuracy assessment.^28^ Targeted sampling of mismatched and matched tumor subregions may provide an opportunity to establish more robust histopathologic and molecular correlates.

T2FMM has been shown to boost the ability of models predicting the molecular status and risk-stratifying gliomas. These models, which rely on a combination of clinical, qualitative radiologic, or radiomic features and are either based on multivariate logistic regression or machine learning, require further validation.^44–51^

Moreover, the suggested association of “fading” T2FMM upon adjuvant treatment of incompletely resected tumors remains poorly characterized and understudied, despite the exciting potential for using the sign as a treatment monitoring tool.^15^ Similarly, initial studies exploring the potential uses of T2FMM for entities other than adult-type diffuse gliomas, such as pediatric-type diffuse high-grade gliomas and DNETs, have yielded promising results, thus encouraging further research in this direction.^27,28,30^

## Conclusion

T2FMM is a recently defined radiogenomic sign characterized by near-perfect specificity, yet relatively low sensitivity, for the diagnosis of IDHm non-codeleted astrocytomas in adults. This powerful imaging tool allows risk-stratification of gliomas based on noninvasive and preoperative prediction of their molecular subtype, thus improving patient counseling and presurgical planning. Due to inherently subjective criteria, the definition of this sign can be “relaxed” resulting in a “partial” or “modified” mismatch. The latter application compromises its specificity for the 1p/19q non-codeleted, but not to the IDHm phenotype, allowing clinicians and researchers to confidently rule-out the more aggressive IDHwt phenotype. This heterogeneous application, as well as its use in pediatric-type CNS tumors, constitutes the source of most “false positive” cases in the neuroradiology literature. Histologic analyses have attributed the FLAIR suppression to the presence of large intercellular spaces, including microcysts, at the core of the tumor compared to the highly cellular periphery. Mismatch-positive gliomas also showed upregulated mTOR pathway expression, higher ADC, and lower rCBV compared to their mismatch-negative counterparts. However, more elaborate studies are required to better characterize the latter associations, among others, relevant to this powerful radiogenomic sign.
